# Consider autoimmune encephalitis in cases of headache, dizziness, and mild pleocytosis following SARS-CoV-2 infection

**DOI:** 10.1186/s42466-026-00469-5

**Published:** 2026-02-09

**Authors:** Sinda Zarrouk, Josef Finsterer

**Affiliations:** 1 Institute Pasteur of Tunis, University of Tunis El Manar and Genomic Platform, Tunis, Tunisia; 2 Neurology Department, Neurology & Neurophysiology Center, Postfach 20, Vienna, 1180 Austria

Letter to the Editor,

We read with interest the article by Umathum et al. about a 75-year-old female patient with holocranial headache, dizziness, upper extremity paraparesis, and glove-like sensory disturbances following a SARS-CoV-2 infection (SC2I) seven weeks prior [1]. Diagnostic workup revealed tetraparesis, axonal neuropathy, an older ischemic lesion in the cuneus, atrial thrombosis, and mild pleocytosis unresponsive to prednisolone [1]. Twelve days after admission, the patient died suddenly. Autopsy revealed acute myocardial infarction without significant coronary stenosis, cerebral T-lymphocytic infiltration, and necrotizing inflammatory myopathy of the diaphragm [1]. The study is promising, but some points require further discussion.

The first point concerns the discrepancy between the statement in the abstract that the patient showed no central nervous system (CNS) involvement during his lifetime and the case description, which mentions that the patient complained of headaches and dizziness seven weeks after SC2I [1]. Since holocranial headaches and dizziness are clearly CNS symptoms, this discrepancy should be clarified.

The second point concerns the patient’s mild pleocytosis. However, the search for infectious causes and vasculitis was inconclusive [1]. Regarding infectious causes, it is important to know which pathogens the cerebrospinal fluid was tested for. Viruses that can cause encephalitis include herpes simplex virus, Epstein-Barr virus, chickenpox, West Nile virus, Zika virus, equine encephalitis, enterovirus, adenovirus, cytomegalovirus, measles, mumps, rubella, rabies, and Japanese encephalitis. Other infectious causes include Lyme disease, syphilis, tuberculosis, fungal infections, toxoplasmosis, and roundworm infections, especially in immunocompromised patients [2]. Were all these pathogens truly ruled out as the cause of the encephalitis?

The third point is that the cerebral MRI was performed without contrast. Encephalitis can easily be missed when multimodal MRI is performed without contrast [3].

The fourth point is that the patient was not tested for autoimmune encephalitis [1]. Since autoimmune encephalitis has been described as a complication of SC2I [4], it would have been essential to test cerebrospinal fluid and serum for AIE-associated antibodies.

The fifth point concerns the left atrial thrombus [1]. The cause of the thrombus formation in the left atrium should be clarified. Was atrial fibrillation ever documented on a standard or Holter ECG? Was atrial fibrillation the cause of the ischemic stroke visible on cerebral MRI? Was there evidence of myocarditis, endocarditis, or pericarditis, which have been described as complications of SC2I [5]? Did the patient suffer from a coagulation disorder? Was the atrial thrombus the cause of the myocardial infarction?

The sixth point concerns SUDEP, which was not considered as a cause of sudden unexpected death [1]. Was the postmortem creatine kinase (CK) level elevated? Sudden unexpected death can also be caused by Takotsubo syndrome or neurogenic pulmonary edema. What was the size of the infarct at autopsy?

In summary, infectious or autoimmune encephalitis following SC2I infection can easily be overlooked if not actively sought. Sudden cardiac death in a patient with a myocardial infarction is not necessarily due to a coronary artery occlusion.

## Data Availability

All data are available from the corresponding author.
